# Application of Ice Temperature Storage Technology Assisted by Chlorine Dioxide and Chitosan for the Preservation of Fresh Fish Slices

**DOI:** 10.1002/fsn3.70127

**Published:** 2025-04-21

**Authors:** Wenping Yang, Kunyu Sui, Fawei Qiu, Qinhuizi Zhu, Jianlin Luo, Shirui Yu

**Affiliations:** ^1^ Department of Food Science and Engineering Moutai Institute Renhuai China; ^2^ College of Biological and Environmental Engineering Guiyang University Guiyang China; ^3^ Guizhou Health Wine Brewing Technology Engineering Research Center Moutai Institute Renhuai China

**Keywords:** chitosan, chlorine dioxide, fish preservation, ice temperature storage, sturgeons

## Abstract

To solve the problem of fresh fish and meat being prone to spoilage and deterioration during storage and transportation, thus causing great economic losses, we investigated the efficacy based on the synergistic antibacterial effect of chlorine dioxide and chitosan. This paper developed an ice temperature storage technology for the preservation of fresh sturgeon fillets. The research results showed that among the five common different freezing point regulators, only NaCl, glucose, and sucrose displayed negligible effects on the texture of fish slices. The optimal ratio obtained through response surface optimization was 3.07 g/100 mL NaCl, 3.25 g/100 mL glucose, and 4.05 g/100 mL sucrose, which could lower the freezing point of fish fillet to −2.50°C. Based on the changes in volatile base nitrogen, thiobarbituric acid reactants, pH value, and total viable bacteria during storage, the spoilage endpoints of sturgeon fillets stored at ice temperature (−2°C) and low temperature (4°C) were determined to be 9–12 days and 3–6 days, respectively. Based on high‐throughput sequencing and traditional culture‐isolation techniques, four dominant spoilage bacteria were successfully isolated and identified during stored at ice temperature, including *Chryseobacterium* sp., *Microbacterium* sp., *Empedobacter falsenii*, and *Bacillus cereus* . For the three components (chitosan, sodium alginate, and poly‐L‐lysine HCl) of the coating, the response surface optimization results showed that the optimal fresh‐keeping ratio was 2.12 g/100 mL of chitosan, 0.72 g/100 mL of sodium alginate, and 1.02 g/100 mL of poly‐L‐lysine HCl. Under optimal storage conditions, the TVB‐N, TBARS, pH, and other parameters in fish meat would be significantly decreased.

## Graphical abstract

Based on the synergistic antibacterial effect of chlorine dioxide (ClO_2_) and coating, this paper developed an ice temperature storage technology assisted by ClO_2_ and coating for the preservation of fresh sturgeon fillets. The storage technology could significantly reduce the total amount of microorganisms in fish slices maintain their nutritional value, extending its shelf life to 15 days.

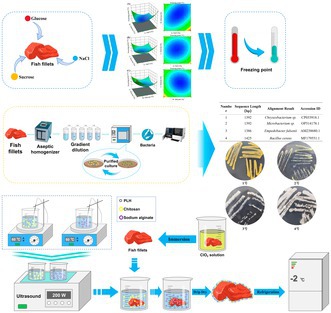

## 1 Introduction

Sturgeons is a large and medium‐sized sub cold water freshwater fish and is one of the most common aquaculture fish in the market, which can directly create huge economic benefits every year. China is a major sturgeons aquaculture country in the world, accounting for approximately 86% of global production (Yuan et al. 2024). Sturgeon is highly favored by consumers due to its rich nutrition, delicious meat, lack of spines, and absence of fishy smell (Lin and Chen 2024). Especially, sturgeon is rich in various proteins, amino acids, fatty acids, and mineral elements (Hung 2017). Therefore, sturgeon fillets are easily infected by microorganisms, which accelerates spoilage and deterioration. Currently, refrigeration and frozen storage are the most popular storage methods for fresh fish meat. Traditional refrigeration involves storing fish meat at a temperature of 4°C, which can inhibit microbial activity to a certain extent without damaging the cells of the fish meat, thus preserving its quality in the short term. However, due to the incomplete inhibition of microorganisms and internal reactions of fish meat in this situation, the storage time is usually only about 2 days. On the contrary, frozen storage (~ −20°C) can almost inhibit the activity of microorganisms and fish meat itself, thus achieving a longer shelf life. However, there are some limitations in this method, such as drip loss, protein denaturation, and diminished product quality caused by damage from ice crystal formation (Jiang et al. 2024). Especially during the thawing process, there is a greater loss of nutrients. Ice temperature preservation, a preservation method between refrigeration and frozen storage, has attracted people's attention. Ice temperature preservation is a novel approach to fresh food preservation involving temperature control ranging from 0°C to the freezing point (Jiang et al. 2024). It combines the advantages of refrigeration and frozen storage while avoiding their drawbacks. This technology has begun to assist in the preservation of fruits, vegetables, meat, and shrimp (Sun et al. 2020; Liu et al. 2022, 2020; Wang et al. 2015).

Usually, a single preservation technique cannot achieve the best preservation effect, while composite preservation techniques are more effective in most cases. Chitosan (CS), a natural amino polysaccharide, can be obtained through the deacetylation reaction of chitin (Wang et al. 2020). It is rich in sources, highly biocompatible, non‐toxic, biodegradable, and film‐forming, as well as has antioxidant, antibacterial, and antifungal properties (Li et al. 2023; Lan et al. 2022). Therefore, CS‐based bio‐coatings have attracted much attention in the field of food preservation. For example, CS coating preservation was proven to not only significantly inhibit the growth of mold and yeast, but also reduce the activity of polygalacturonase and pectin methyl esterase, as well as better maintain the color and hardness of fresh cut papaya. As a result, it reduced the deterioration process of fresh cut papaya stored at 5°C, maintained its quality, and extends the shelf life (González‐Aguilar et al. 2008). ClO_2_ is a broad‐spectrum, highly efficient, safe, and low‐cost antibacterial agent that has been classified as Class A1 disinfectant by the World Health Organization and the Food and Agriculture Organization of the United Nations (Zhu et al. 2024). It can penetrate the cell membrane of microorganisms, disrupt their osmotic pressure, affect their enzymes, interfere with their biosynthesis, and ultimately lead to microbial death (Zhao et al. 2022). Within a wide pH range (3.0 ~ 8.0), it has significant antibacterial effects on bacteria, fungi, yeast, molds, viruses, etc. (Kessler et al. 2023). In addition, it also has the characteristics of strong oxidation ability, safety without residue, and no toxic by‐products. Especially, it does not alter the nutritional and sensory quality of food and fresh agricultural products (Sun et al. 2019). Therefore, ClO_2_, as a new generation of preservatives and fungicides, has been applied to the packaging and disinfection of fresh meat, meat products, and other agricultural products (Singh et al. 2021). For example, Li et al. (Li et al. 2022) studied an instantaneous treatment method using ClO_2_ and heat. It was found that treatment with ClO_2_ gas for 10 min significantly inhibited the *X. campestris* population on cabbage, chili pepper, and radish seeds, as well as the initial population of *S. entericas* on cabbage and pepper seeds.

In this paper, based on the ice temperature preservation and the antibacterial effect of ClO_2_ and CS, an ice temperature preservation technology was studied for the preservation of fresh fish fillets. Firstly, the effects of different freezing point regulators on the freezing curve, texture, and pH of sturgeon were studied to obtain suitable freezing point regulators. The optimal concentration of freezing point regulator was obtained by response surface optimization method. Subsequently, high‐throughput sequencing and traditional culture‐isolation technology were used to analyze the composition, structure, and abundance of microorganisms during storage, and their changes were studied. Finally, we explored the fluctuations of specific key factors under different treatment conditions, including TVB‐N, TBARS, and pH. This work provides some key factors for the storage and preservation of sturgeon.

## 2 Materials and Methods

### 2.1 Materials and Chemicals

Sturgeon was obtained from the local market. Sodium chloride (NaCl), calcium chloride (CaCl_2_), glucose, sucrose, and vitamin C (VC) were provided by Sinopharm Chemical Reagent Co. Ltd. (China). Potassium chloride (KCl) was bought from Kemio Chemical Reagent Co. Ltd. (Tianjin). CS (deacetylation degree 95%) and alginate were supplied by Macklin Biochemical Co. Ltd. (China). Poly‐L‐lysine HCl (PLH) (food grade) was come from Yinuo Biotechnology Co. Ltd. (Zhejiang). Chlorine dioxide disinfectant powder (food grade) was bought from Hualong Xingyu Technology Development Co. Ltd. (Beijing).

### 2.2 Determination of the Freezing Point

The purchased sturgeon was transported to the laboratory in vivo. After being knocked out, the head, fin, skin, and viscera were removed. The remaining fish meat was rinsed with sterile water, divided into fish pieces with a size of 2 × 2 × 2 cm, and put into sterile fresh‐keeping bags. As shown in Figure S1, sturgeon trunk muscles were divided into four parts: front (A), middle (B), rear, (C), and tail (D). After the samples of different parts were put into the refrigerator (−20°Ca), the probe of the temperature recorder was inserted into the center of the samples at the position of 1 cm and set to record the temperature change in the center of the samples every 1 min; according to the recorded data, a temperature change curve was plotted. After analysis, the freezing point of each part of the fish body was determined.

### 2.3 Preparation of Coating Solution and Treatment of Fish Slices

Preparation of coating solution: Coating solution A: A certain amount of CS was weighed and dissolved in 100 mL of 1% acetic acid solution, with 1 mL of glycerol and a certain amount of PLH, and then heated and stirred at 60°C for 24 h until it was completely dissolved. Subsequently, 200 W ultrasound was used to remove bubbles, resulting in a CS composite coating solution. Coating solution B: A certain amount of sodium alginate was weighed into a beaker, after adding 100 mL of deionized water, heated, and stirred it at 60°C for 24 h until it was completely dissolved. After removing bubbles with 200 W ultrasound, the obtained liquid was sodium alginate coating solution. Coating treatment of sturgeon slices: Sturgeon slice was immersed in the coating solution A for 2 min and removed and drained until no more solution drips. Then it was put into coating solution B for 2 min; after it was taken out and drained, the sample was placed in a sterile culture dish.

### 2.4 Chlorine Dioxide Treatment of Fish Fillets

Fresh sturgeon slices were treated with chlorine dioxide solutions of different concentrations (20, 30, 40, 50, and 60 mg/L) for 1, 3, 5, 7, and 9 min, respectively. The surface solution was washed with sterile water, and the relevant parameters were measured.

### 2.5 Determination of Texture

The texture of fish meat was analyzed by a texture analyzer (Universal TA, Shanghai tengba Instrument Technology Co. Ltd). Parameter setting: With the P/36R probe, the test speed was 2 mm/s at the beginning, 1 mm/s in the test, and 5 mm/s at the end. The compression ratio of fish meat reached 50%, and the pressure residence time was maintained for 5 s. Exporting data from the instrument.

### 2.6 Determination of pH

10 ± 0.5 g of minced fish meat was weighed and put into a 50 mL beaker. After adding 10 mL of 0.5 mol/L KCl solution, it was homogenized via a homogenizer (Ultra Turrax T18, IKA Group, Germany) at 5000 rpm for 2 min, and then the pH value was measured by the pH meter (ST3100, Changzhou Ohaus Instrument Co).

### 2.7 Determination of Key Parameters

In the ultra‐clean workbench, the fresh sturgeon slices were treated differently, then put into sterile Petri dishes, and placed in a 4°C or − 2.0°C refrigerator for storage. The samples were taken out at 0, 3, 6, 9, 12, and 15 days, and the total number of microorganisms (GB 4789 2–2022 2022), pH value, TVB‐N (GB 5009 228–2016 2016), and TBARS (GB 5009 181–2016 2016) was detected. The details of the process are as follows.

The total number of colonies of tissue samples at different moments was determined, and TVC was calculated using a PCA plate counting medium according to the Chinese standard GB4789.2–2022 for the microbiological examination of food in food safety. 5 g of the sample was weighed in a sterile bag, and 45 mL of sterile saline was added and aseptically homogenized for 2 min to obtain a 10‐fold diluted sample solution. It was continued to dilute to a certain gradient, 1 mL of the appropriate dilution solution coated in PCA medium was taken, with incubation at 30°C for 48 h, and the number of colonies in the 30–300 range was selected between the plate as the effective plate, with the unit of colony counting with each gram of the sample colony‐forming units expressed (log_10_CFU/g).

Determination of total volatile base nitrogen (TVB‐N) was according to the second method of China's national food safety standard GB5009.228–2016 automatic Kjeldahl method, using a Kjeldahl analyzer Kjeltec (KDN812, Shanghai Xianjian Instrument Co). TVB‐N results were expressed in mg TVB‐N per 100 g.

Determination of thiobarbituric acid reactive substances (TBARS) was according to the method of China's national food safety standard GB5009.181–2016 Determination of malondialdehyde in foodstuffs. The fish was homogenized and homogenized, extracted with 7.5% trichloroacetic acid, and then reacted with 0.02 mol/L thiobarbituric acid to form a pink complex under the condition of water bath at 95°C, and the absorbance at 532 nm was determined for each reaction sample. The standard curve was plotted with malondialdehyde standard (1.1.3.3‐tetraethoxypropane).

### 2.8 High Throughput Gene Sequencing Analysis

5 g of the sample was taken, and its DNA was extracted for PCR amplification. The amplification primers were as follows: 338F: 5'‐ACTCCTACGGGAGGCAGCAG; downstream primer: 806R: GGACTACH VGGGTWTCTAAT‐3′; the PCR reaction system is shown in Table 1. PCR reaction parameters were as follows: 95°C for 3 min; (95°C for 30 s; 55°C for 30 s; 72°C for 45 s) × cycle number 27; extension at 72°C for 10 min; it was stored at 10°C until use. PCR products were detected via 2% agarose gel electrophoresis.

**TABLE 1:** PCR reaction system.

Reagent	Dosage
2 × Pro taq mix	10.0 μL
Forward primer (5 μM)	0.8 μL
Reverse primer (5 μM)	0.8 μL
Template DNA	10 ng
Distilled water	Add to 20.0 μL

### 2.9 Isolation, Purification, and Identification of Microorganisms

For isolation and purification, refer to the previous report (Tshabalala et al. 2021) with slight adjustments. Briefly, in the ultra‐clean environment, 5 g of fish meat was put into a sterile homogenization bag with 45 mL of normal saline. After mixing it well, it was homogenized for 2 min via a sterile homogenizer. Then 1 mL of the homogenate was diluted five times with saline according to a 10‐gradient. A certain amount of dilution solution was coated on the medium of PCA, TSA, LB, TSA, etc. and then put into a 34°C incubator for culture. Single colonies exhibiting good growth were carefully selected and transferred into LB medium. The isolated strains were then purified for 2 to 3 generations. Subsequently, observation and photography were carried out to record the colony morphology.

Amplification and identification: The DNA extracted from a single spoilage microorganism was used as the template for PCR amplification and identification (the primer sequence was 27F: 5′‐AGAGTTTGATCCTGGCTCAG, 1492R: GGTTACCTTGTTACGACTT‐3′). The PCR reaction system is shown in Table 1. PCR reaction parameters were as follows: 95°C for 5 min; (95°C for 30 s; 56°C for 30 s; 72°C for 90 s) × cycle number 25; extension at 72°C for 10 min. Sanger sequencing was used to detect the PCR products.

### 2.10 Data Processing and Analysis

SPSS 27.0 was used to conduct one‐way analysis of variance (ANOVA) on the data, and correlation analysis was conducted on the statistically significant data, with *p* < 0.05 as the significant level of difference. Data processing was performed with origin2019 software. All measurements were performed at least in triplicate.

## 3 Results and Discussion

### 3.1 Creation and Analysis of Freezing Point Adjustment Programs

The ice zone of food is usually very narrow, which often makes it difficult to control the temperature in the ice preservation process. Introducing freezing point regulators is a commonly used method to address this issue. Many freezing point regulators have been reported, and here we will study the ice temperature preservation of sturgeon using five common ones. Prior to this, we first measured the freezing point of sturgeon, as shown in Figure S2. The freezing points of the front (A), middle (B), back (C), and tail (D) of sturgeon were − 1.2°C, −1.2°C, −1.1°C, and − 1.1°C, respectively. The freezing points of various parts were basically the same, and it could be determined that the freezing point of sturgeon was −1.2°C. To investigate the effect of different freezing point regulators on the freezing point of sturgeon, we added different concentrations of NaCl, CaCl_2_, glucose, sucrose, and vitamin C. The freezing curves of sturgeon in the presence of different freezing point regulators are displayed in Figure S3. When NaCl solutions with concentrations of 1.0, 2.0, 3.0, 4.0, and 5.0 g/100 mL were added, the freezing points were − 1.6°C, −1.7°C, −2.1°C, −2.0°C, and − 1.9°C, respectively. The freezing point adjustment range was −1.6°C ~ −2.1°C (A). Similarly, with different concentrations of CaCl_2_ solutions, the freezing points were − 1.6°C, −1.5°C, −1.7°C, −1.8°C, and − 1.9°C, respectively. In this case, the freezing point adjustment range was −1.5°C ~ −1.9°C (B), which was much lower than −1.2°C. Treating with different concentrations of glucose solutions, the freezing points were − 1.6°C, −1.6°C, −2.0°C, −1.7°C, and − 1.6°C, respectively, and the freezing point adjustment range was −1.6°C ~ −2.0°C (C). On introducing a series of sucrose contents, the freezing points were − 1.7°C, −1.8°C, −1.8°C, −1.9°C, and − 1.6°C, respectively, where the freezing point adjustment range was −1.6°C ~ −1.9°C (D). Once different concentrations of vitamin C (0.25, 0.50, 0.75, 1.00, and 1.25 g/mL) were added, the freezing points obtained were − 1.7°C, −1.8°C, −1.5°C, −1.9°C, and − 1.7°C, respectively, and the freezing point adjustment range was −1.5°C ~ −1.9°C (E). It was obvious that the freezing point range had been expanded, and the obtained freezing points were significantly lower than the control group (−1.2°C).

**FIGURE 1:**
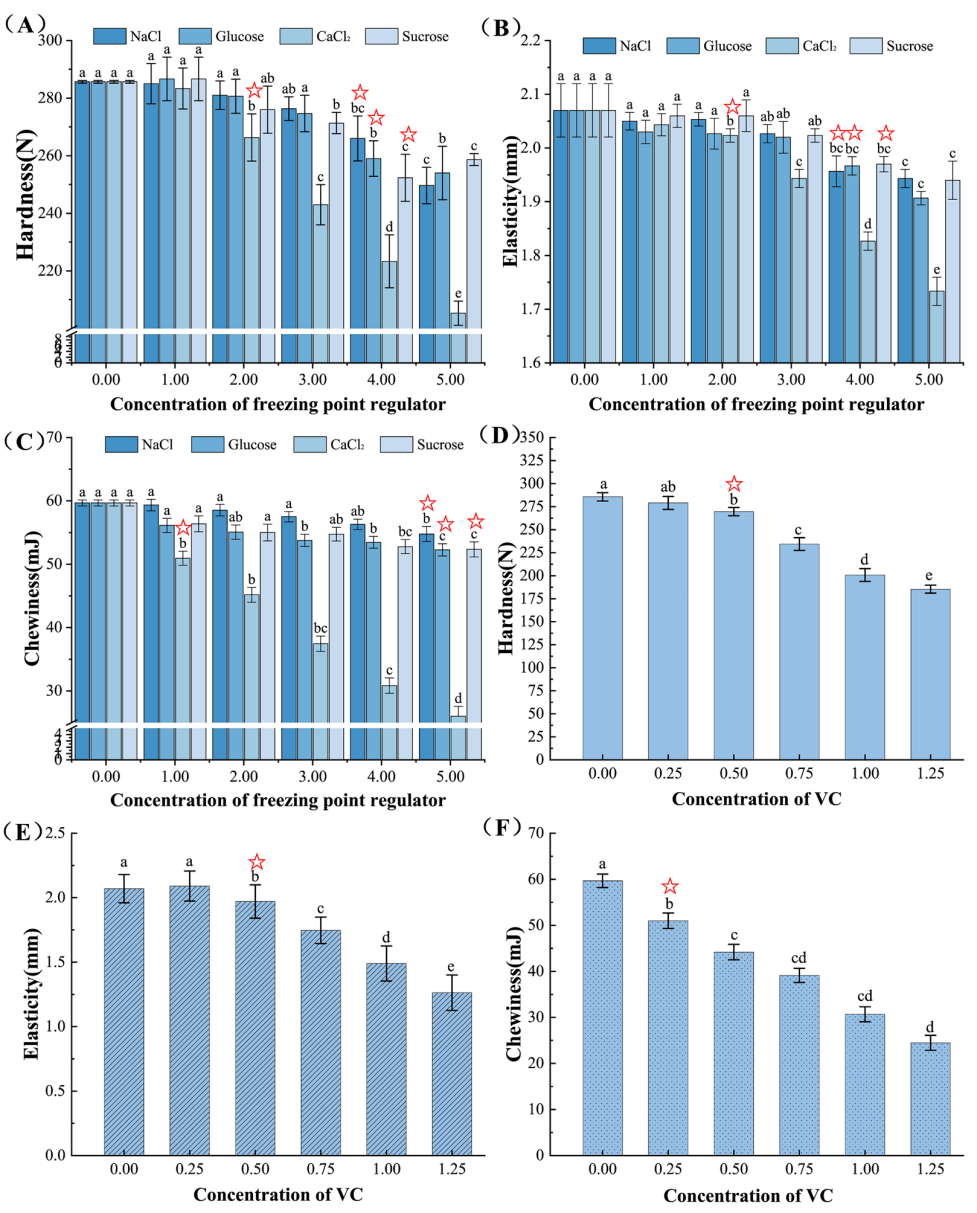
Effect of freezing point regulators with different concentrations on the hardness (A, D), elasticity (B, E), and chewiness (C, F) of sturgeon.

The addition of freezing point regulators can sometimes have an impact on the sensory perception of food, for which we conducted a texture assessment. It could be observed that low concentrations of freezing point regulators had little effect on the sensory quality of sturgeon, but their quality would decrease to varying degrees as the concentration of freezing point regulators increases (Figure 1). The results showed that when the treatment concentration of NaCl, glucose, and sucrose increased to 4.0 g/100 mL, the hardness and elasticity of sturgeon significantly decreased (*p* < 0.05); when the concentration of CaCl_2_ solution was only 2.0 g/100 mL, the hardness and elasticity had already significantly decreased (*p* < 0.05). For chewiness, NaCl had the least impact on sturgeon chewiness, with a significant decrease observed only at a concentration of 5.0 g/100 mL (*p* < 0.05), followed by the glucose and sucrose solution treatment groups. The chewiness of sturgeon in the CaCl_2_ treatment group was significantly affected, with a significant decrease observed at a concentration of 1.0 g/100 mL (*p* < 0.05). When different vitamin C was added for treatment, the hardness and elasticity of sturgeon were significantly reduced in the 0.5 g/100 mL group (*p* < 0.05), while the chewiness was significantly reduced in the 0.25 g/100 mL group (*p* < 0.05). It is speculated that this may be caused by the low pH properties of vitamin C, as a low pH environment can easily affect the three‐dimensional structure of proteins, thereby losing their original physical and chemical properties (Jankowiak et al. 2021). It could be confirmed that the texture of sturgeon was greatly influenced by CaCl_2_ and vitamin C, even at lower concentrations; in contrast, the treatment of NaCl, glucose, and sucrose had no significant effect on the texture of sturgeon at certain concentrations, making them more suitable as freezing point regulators for sturgeon. To further investigate, we investigated the interference of freezing point regulators on the pH value of fish meat (Figure S4A–D). It could be observed that the treatment groups with different concentrations of NaCl, CaCl_2_, glucose, and sucrose solutions had no significant effect on the pH of sturgeon (*p* > 0.05). Their pH fluctuated within the ranges of 5.93 ~ 5.98, 5.74 ~ 5.80, 5.91 ~ 5.94, or 5.89 ~ 5.93, respectively, and there was no significant difference between the concentrations. However, Figure S4E shows that the pH of sturgeon remained in a decreasing trend as the increased concentration of vitamin C, and there were significant differences between the concentrations (*p* < 0.05). This further confirms our speculation mentioned above. In summary, NaCl, glucose, and sucrose are more suitable for ice temperature preservation of sturgeon among the five common freezing point regulators.

**FIGURE 2:**
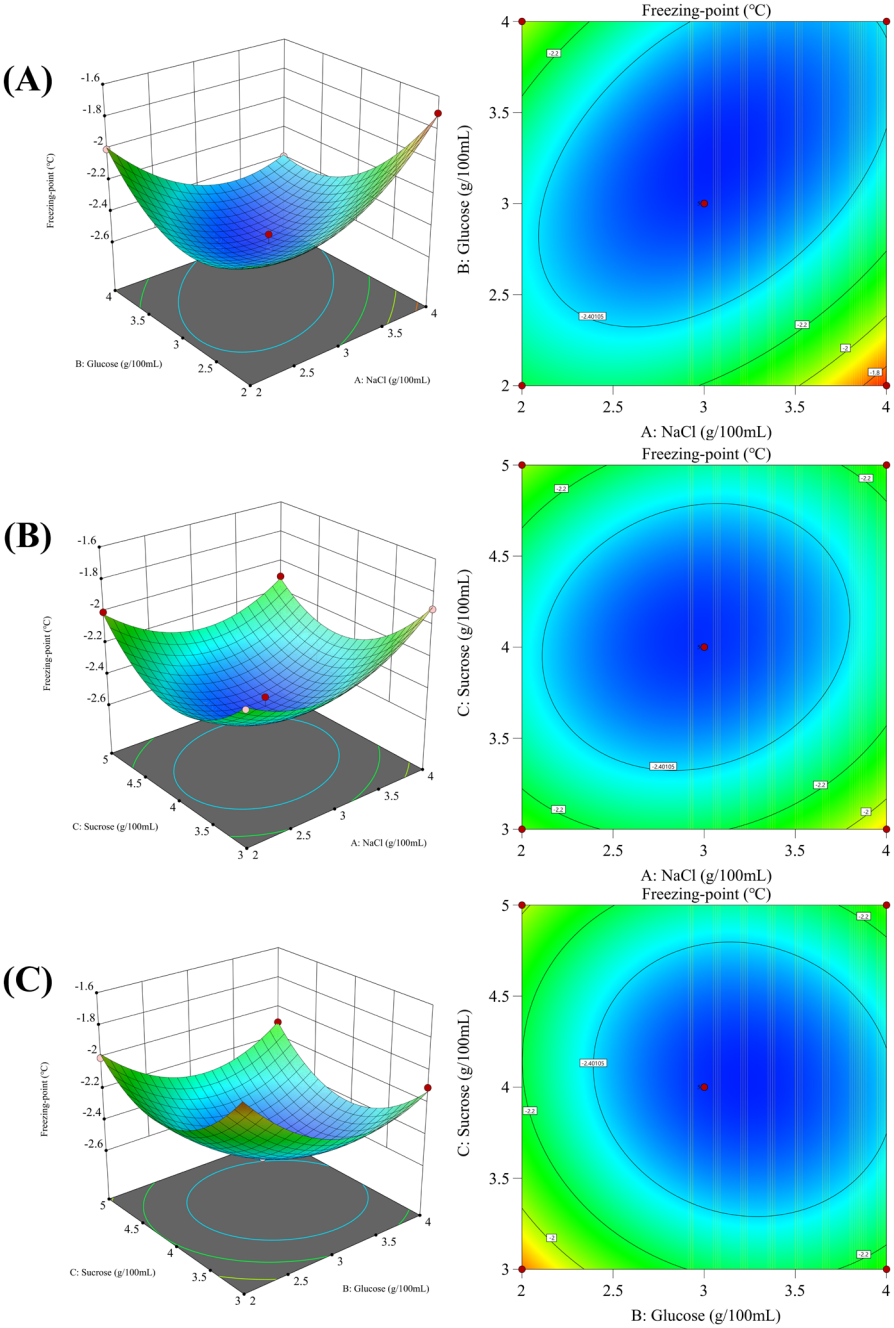
Response surface and contour map of the interaction effect of related combinations on freezing point. (A) NaCl and glucose. (B) NaCl and sucrose. (C) Glucose and sucrose.

To better achieve ice temperature preservation of sturgeon, we subsequently optimized the three freezing point regulators mentioned above. The results of single factor optimization showed that the lowest freezing point temperature could be obtained when the concentrations of NaCl, glucose, and sucrose were 3.0 g/100 mL, 3.0 g/100 mL, or 4.0 g/100 mL, respectively (Figure S5). Table S1 presents the results of response surface optimization experiments. Regression analysis was conducted on the data in Table S1 using the design‐expert13 software. The quadratic multiple regression model between the freezing point of sturgeon and the concentrations of three regulators was obtained as follows: *Y* = −2.56 + 0.0250A−0.1125B−0.0375C−0.2250AB−0.0750 AC + 0.0500 bc + 0.2300A^2^ + 0.2550B^2^ + 0.3050C^2^. The variance analysis has been conducted on the model, and the results obtained are shown in Table S2. The model had significance (*p* < 0.001), while the omission item was not significant (*p* = 0.8395 > 0.05), *R* ^2^ = 0.9893, *R* ^2^_Adj_ = 0.9756. This indicates that the regression equation had a high reliability that could be applied to analyze and predict the effects of three regulators on the regulation of freezing point. According to the above results, the obtained response surface and contour map are shown in Figure 2. The results showed that the interaction between NaCl and glucose as well as NaCl and sucrose on the freezing point of sturgeon was significant (*p* < 0.001), which had a significant impact on the freezing point. The optimal concentration of freezing point regulator was determined to be 3.075 g/100 mL of NaCl, 3.248 g/100 mL of glucose, and 4.051 g/100 mL of sucrose, with a predicted freezing point of −2.57°C. Actual experiments further verified that this formula could lower the freezing point of sturgeon to −2.5°C. At the same time, the three substances in the formulation affected the osmotic pressure regulation mechanism within the sturgeon cells, making the intracellular water less prone to freezing at low temperatures. Further studies showed that this solution not only prolonged the freshness period of sturgeon fillets under ice temperature storage conditions but also had no significant negative impact on the quality of sturgeon such as taste and color. Compared with untreated sturgeon fillets, the treated sturgeon was able to maintain better muscle elasticity and freshness at −2.5°C, and the growth rate of spoilage indicators such as TVB‐N was significantly slowed down, thus providing an effective technical means for low‐temperature storage and preservation of sturgeon.

### 3.2 Development of New Ice Temperature Preservation Technology

ClO_2_ is highly favored preservatives. We used it to assist in the ice temperature preservation of sturgeon to achieve the best possible preservation effect. Usually, its treatment concentration is crucial for preservation effectiveness. For ClO_2_, Figure S6 displays that the bacterial count of sturgeon slices was influenced by both treatment time and ClO_2_ concentration. Under the same concentration of ClO_2_ treatment, the bacterial count in fish gradually decreased with the extension of treatment time. When lower concentrations of ClO_2_ were applied (20 ~ 40 mg/L), even if the treatment time was extended (9 min), its inhibitory effect on microorganisms was not very significant. However, when the concentration of ClO_2_ was increased, even within a short period of time (5 min), microorganisms were maximally killed. Therefore, 50 mg/L of ClO_2_ was chosen as the subsequent treatment concentration. In addition, the optimization of preservative components in the film is essential to obtain better preservation effect. The TVB‐N value can be used as a key parameter of fish spoilage, because TVB‐N is closely related to the activity of microbial life. Microbial proteases degrade proteins in fish bodies, and fish spoilage leads to the rise of TVB‐N content. Figure S7A shows the change of TVB‐N value of sturgeon treated with coating solution with different concentrations of CS. With the increased concentration of CS, the TVB‐N value of sturgeon slices decreased until the CS concentration reached 2.0 g/100 mL. Therefore, 2.0 g/100 mL was selected as the optimal concentration of CS. Figure S7B shows the effect of different concentrations of sodium alginate on the storage quality of sturgeon slices. The content of TVB‐N gradually decreased with the increase of sodium alginate concentration. When it reached 0.75 g/100 mL, its TVB‐N content did not change significantly (*p* > 0.05). Therefore, 0.75 g/100 mL was determined as the optimal concentration. It can be seen from Figure S7C that the TVB‐N of sturgeon slices tended to decrease with the increase of PLH content. At a lower concentration of PLH (0.5 ~ 1.5 g/100 mL), the TVB‐N of sturgeon slices decreased rapidly with the change of concentration (*p* < 0.05). After the concentration reached 1.5 g/100 mL, the change of TVB‐N of fish fillets was no longer obvious even if the concentration continues to increase (*p* > 0.05). Therefore, 1.5 g/100 mL was selected as the optimal concentration of PLH.

**FIGURE 3:**
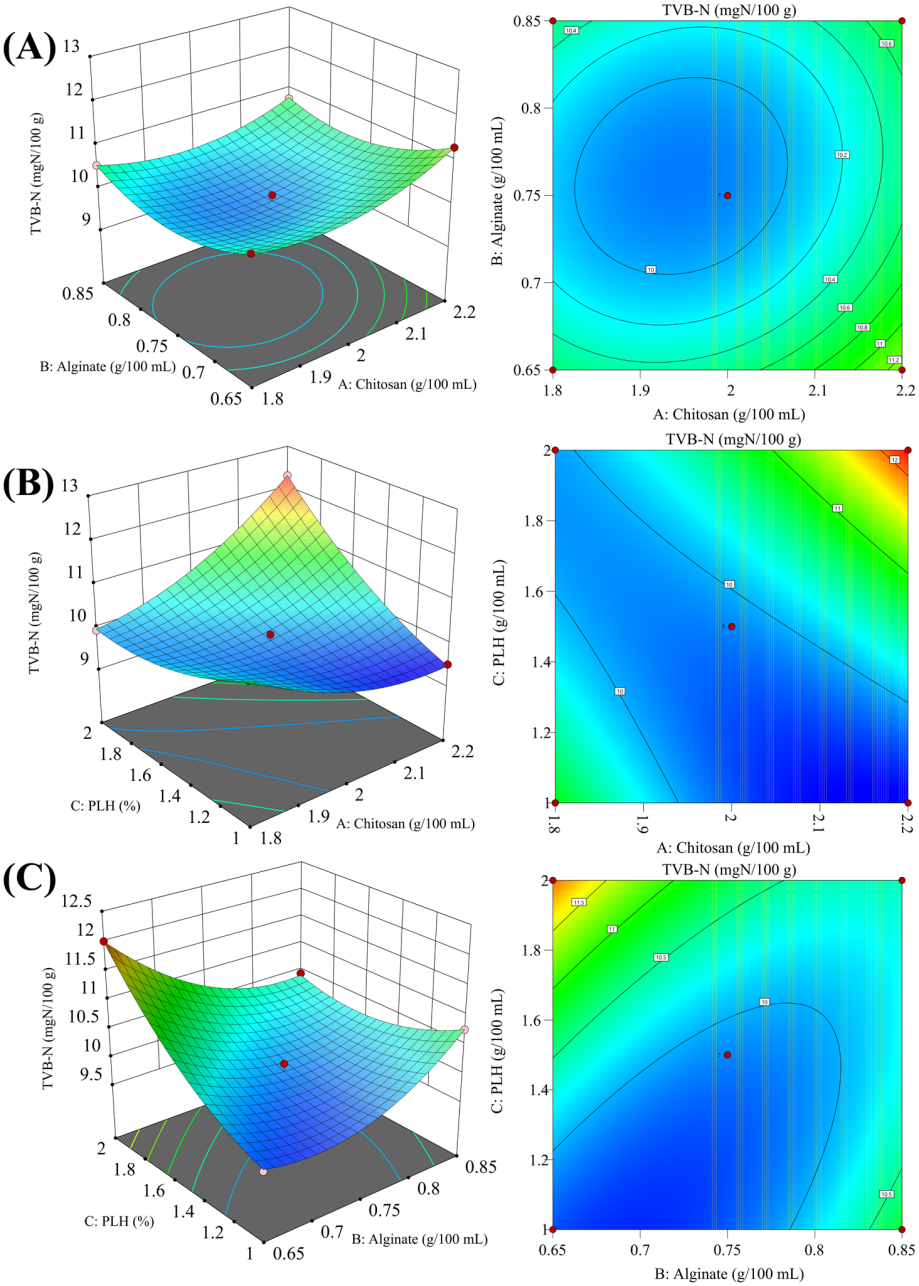
Response surface and contour map of the interaction effect of related combinations on freezing point. (A) Chitosan and alginate. (B) Alginate and PLH. (C) Chitosan and PLH.

To further improve the ice temperature storage effect of the coating solution on sturgeon, we optimized the ratio of each component of the coating solution by response surface methodology based on the above single factor test, and the results are shown in Table S3. Regression analysis was conducted on the data in Table S3 using the design‐expert13 software. The quadratic multiple regression model between the TVB‐N and the concentrations of three preservatives was obtained as follows: *Y* = 9.88 + 0.2368A−0.1409B + 0.4773C−0.1168AB + 0.9326 AC−0.6585 bc + 0.4230A^2^ + 0.4949 B^2^ + 0.3450C^2^. The variance analysis was conducted on the model, and the results obtained are shown in Table S4. According to the results, the model was significant (*p* < 0.001), and the omission item was not significant (*p* = 0.8315 > 0.05), *R* ^2^ = 0.9969, *R* ^2^_Adj_ = 0.9929. It was proved that the fitting degree of the regression equation was very high, and the model could be used to analyze the effect of three preservatives on the preservation of sturgeon slices. According to the above results, the obtained response surface and contour map are shown in Figure 3. The results showed that the primary terms B, AB, and AC of the model had a strong interaction, and the secondary terms A^2^, B^2^, and C^2^ of the model were extremely significant (*p* < 0.01). Therefore, the optimal ratio of preservative was 2.12 g/100 mL of CS, 0.72 g/100 mL of sodium alginate, 1.02 g/100 mL of PLH. After three parallel verifications, it could keep the TVB‐N of sturgeon slices stored for 7 days at 9.56 mg/100 g.

### 3.3 Assessment of Antibacterial Ability

To evaluate the fresh‐keeping ability of this method on fish meat, we first studied its microbial conditions and changes. As shown in Figure S8A, when the 4°C treatment group was stored for 3 ~ 6 days, the microorganism was higher than the maximum safety limit (≤ 6 log_10_ CFU/g) for the total number of fish colonies in the Chinese national standard, while the ice temperature group (only ice temperature treatment) could extend this stage to 6 ~ 9 days. In addition, the total amount and propagation rate of microorganisms in the 4°C treatment group were significantly higher than those in the ice temperature group. Under the condition of ice temperature, we further set up different control experiments, and the results are shown in Figure S8B. Compared with the control group, only ClO_2_ treatment could inhibit the growth of microorganisms to a certain extent in the early stage of storage (0 ~ 12 days), but the level of microorganisms in the late stage of storage was similar with that of the control group. For the coating treatment group without ClO_2_ (Cs + PLH + SA), it did not show excellent antibacterial ability in the early storage (0 ~ 9 days), but showed strong ability in microbial growth and control in the late storage. For the coating treatment group containing ClO_2_, it could significantly inhibit the growth and reproduction of microorganisms on fish fillets during the whole storage stage (including the early and late stages). Therefore, the ClO_2_ and coating assisted ice temperature storage technology proposed in this study has excellent antibacterial effect on fish fillets.

**FIGURE 4:**
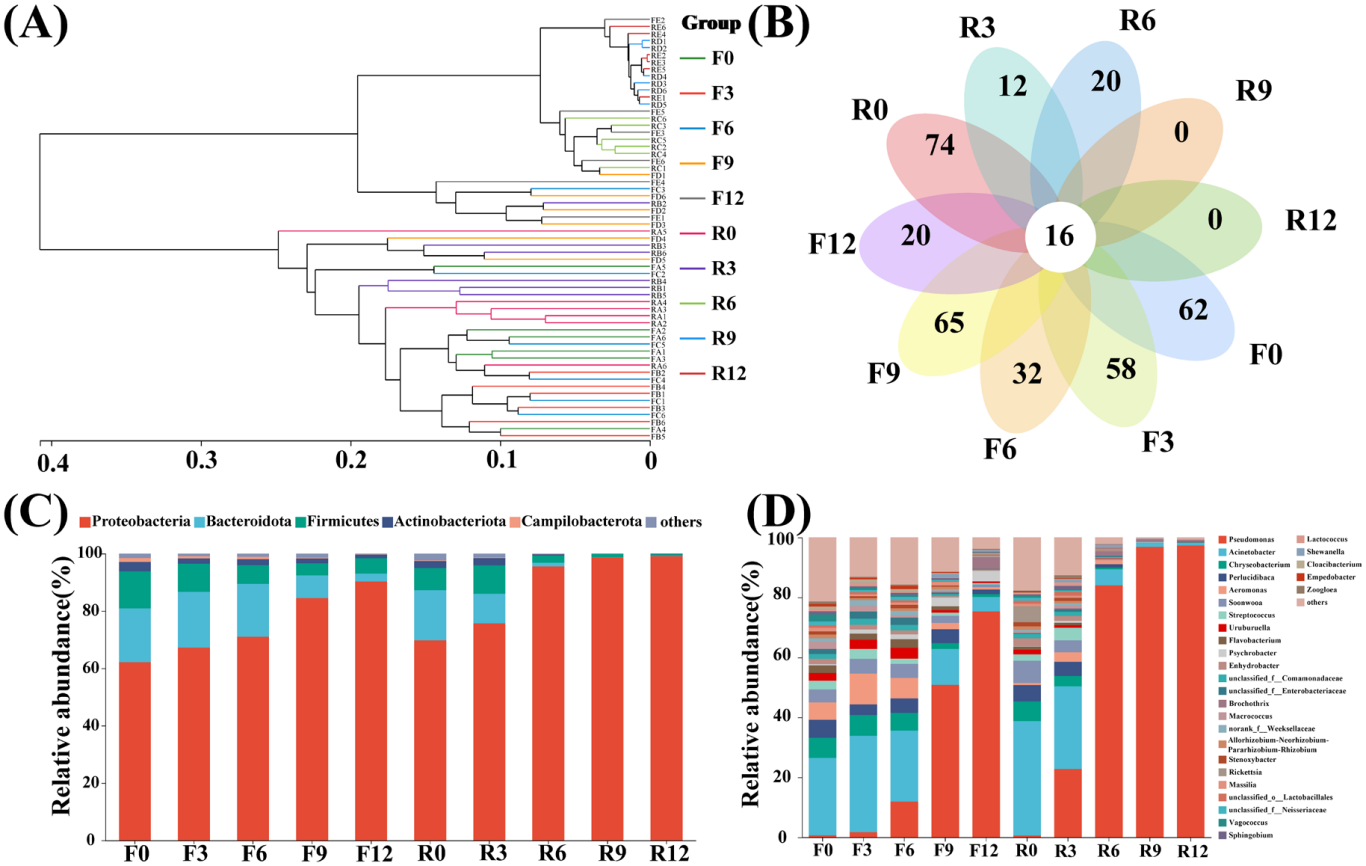
(A) Cluster analysis of microorganisms at the genus level. (B) Veen diagram of the genus of microorganisms. (C, D) The structural composition of bacterial communities at the phylum level (C) and the genus level (D). F_0_, F_3_, F_6_, F_9_, and F_12_ represent the 0th, 3rd, 6th, 9th, and 12th days of ice temperature storage, respectively. R_0_, R_3_, R_6_, R_9_, and R_12_ represent the 0th, 3rd, 6th, 9th, and 12th days of storage at 4°C, respectively.

**FIGURE 5:**
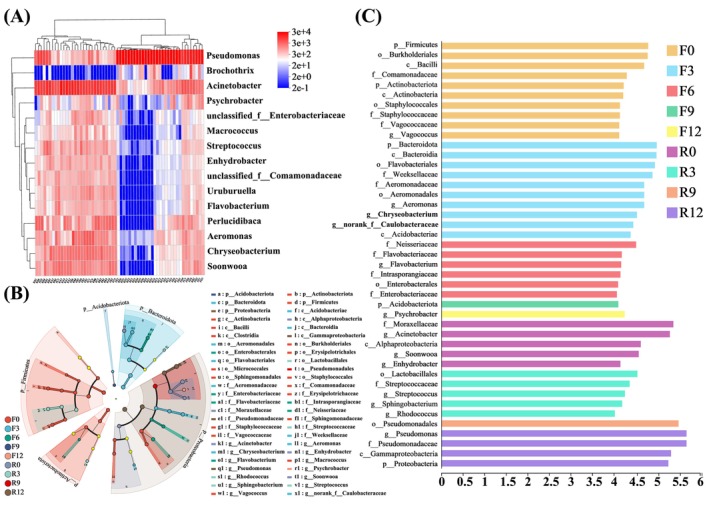
(A) Heatmap of microbial communities. (B) LEfSe analysis of bacterial communities. (C) Linear discriminant analysis of bacterial communities.

Subsequently, high‐throughput sequencing was carried out to further study the antibacterial properties of the preservation technology proposed in this paper. DNA extracted from samples was PCR‐amplified and sequenced. Figure S9 shows the PCR product map of the sample. A single band appeared in both the ice temperature group and the cold storage group, which was suitable for the subsequent sequencing process. Table S5 and Table S6 present the high‐throughput sequencing results of the ice temperature group and the refrigeration treatment group, respectively. Two sets of original sequence data with a total of 60 samples, species annotation database: silva138/16s_bacteria, species annotation method: classify sklarn (Naive Bayes), classification confidence: 0.7, sequence denoising method: DADA2. After optimization, a total of 3,640,492 optimized sequences were obtained, with 1,555,127,369 bases and an average sequence length of 427.05 bp, mainly concentrated in 418 ~ 429. The species annotation results showed a total of 34 phyla, 97 classes, 248 orders, 436 families, and 900 genera. Figure S10A shows the dilution curve of microbial species in the preservation process of sturgeon slices. As the number of observed species increased, the rate of rise of each curve decreased, and the end of the curve tended to be flat, indicating that the measurement data was sufficient and could be used for the next step of microbial analysis. The abundance distribution curve is shown in Figure S10B. The results showed that the microbial composition abundance of sturgeon slices samples was relatively high in the early stage of storage (0–3 days), while the curve decreased rapidly and had a lower width in the later stage of storage (9–12 days), indicating that the abundance and uniformity of the two groups of samples were low currently. This may be related to the nutrients in the fish meat (Li et al. 2019). In addition, the species abundance of the ice temperature group was generally higher than that of the refrigeration group, within the same storage time. It was speculated that compared to the refrigeration environment, microbial activity during the storage of sturgeon slices was further inhibited in the ice temperature environment, and the overall metabolism was lower than that of the refrigeration group. The microbial activity cycle was prolonged, so the species richness within the community decreased slowly. This change pattern is similar with the reported research results (Dourou et al. 2021). Table S6 and Table S7 contain the Alpha diversity analysis indices of the samples, where Chao1 and ACE index are directly proportional to microbial population richness. From the data, although the Chao1 and ACE index of the microbial composition decreased in both the ice temperature group and the refrigeration group, the refrigeration group began to significantly decrease during the mid storage period (6–9 days) (*p* < 0.05), while the ice temperature group could extend the time to 12 d, and the rate of decrease in the refrigeration group was much faster than that in the ice temperature group. The Shannon and Simpson indices can reflect the diversity of microbial composition, where the Shannon index is directly proportional to diversity and the Simpson index is inversely proportional to microbial diversity (Yao et al. 2021). From the table, the Shannon index of the two samples showed a decreasing trend, while the Simpson index showed an increasing trend. Among them, the diversity of the refrigerated group decreased rapidly and was already significant (*p* < 0.05) after 9 days of storage. In contrast, the overall decline of the ice temperature group was relatively slow. It proved that there was a significant change in the microbial community diversity of sturgeon slices in the refrigerated group, while the change was slower in the ice temperature group. For the microbial composition of two treatment groups of sturgeon slices at different time points, hierarchical clustering analysis was conducted at the genus level to obtain Figure 4A. Among them, the R0 and F0 were similar, indicating that the microbial composition of the two groups was similar during the initial storage period. On the 9th and 12th day of storage, the microbial composition of the refrigerated group was comparable, indicating that complete spoilage had occurred on the 9th day, and the spoilage bacteria were *Pseudomonas*. For the ice temperature group, the microbial composition only began to change on the 9th day of storage; especially even after the 12th day (F12), there was still a significant difference compared to the refrigerated group (R12). Subsequently, we used high‐throughput sequencing to analyze the bacterial community structure of fish slices during storage at ice temperature and 4°C, as shown in Figure 4B. Among the 60 samples, there were 62 and 74 core bacterial genera in the ice temperature group and refrigeration group, respectively, during the initial storage period. With the increase of storage time, the number of unique bacterial genera in the ice temperature group gradually decreased, reaching 20 at the 12th day. The change pattern of unique bacterial genera in the refrigeration group was roughly the same as that in the ice temperature group, but the special bacterial genera had completely disappeared on the 9th day. From the perspective of door level (Figure 4C), the ice temperature group and the refrigeration group included *Proteobacteria*, *Bacteroides*, *Firmicutes*, *Actinobacteria*, *Campilobacteria*, etc. The proportion of *Proteobacteria* in the early stage of storage was greater than 60.0%, and it would further increase in the later stage of storage, making it the dominant phylum. In the later stage of storage, compared with the ice temperature group, the refrigerated group mainly consisted of *Proteobacteria* and *Firmicutes*, lacking *Actinobacteria* and *Bacteroidetes*. At the level of subordination (Figure 4D), the dominant bacterial genera in the ice temperature group mainly included *Pseudomonas*, *Acinetobacter*, *Perlucidibaca*, and *Psychobacter*; The dominant bacterial genera in the refrigeration group were *Pseudomonas*, *Acinetobacter*, and *Aeromonas*. Although there were dynamic changes in the genus level microorganisms and a decreasing trend in microbial diversity between the ice temperature group and the refrigeration group, the changes in microbial population diversity under ice temperature conditions were relatively slow and lag. It indicated that the growth of microorganisms was indeed inhibited under ice temperature conditions. Figure 5A shows the microbial heatmap of sturgeon slices in the process of ice temperature and refrigeration preservation. The refrigeration treatment group showed significant fluctuations within 0 ~ 3 d, indicating significant differences in the abundance of microbial communities during the initial stage of storage. The abundance of microorganisms in the ice temperature group fluctuated, but it was lower than that in the refrigerated group. In the later stage, the abundance values of *Pseudomonas* in the ice temperature group and refrigeration group were higher, but the abundance of *Comamonadaceae*, *Uruburuella*, *Flavobacterium*, and *Perlucidibacteria* decreased. There was a significant difference in color compared to the early stage of storage, indicating a significant variation in microbial abundance. It could be confirmed that both the ice temperature group and the refrigeration group have higher microbial composition and abundance in the early stage of storage, while the microbial composition on sturgeon slices tended to be single in the later stage. LEfSe analysis and linear discriminant analysis (LDA) were conducted to determine the microbial communities with significant differences or significant enrichment in each group. 55 bacterial communities with LDA scores greater than 4 and significant differences were found in sturgeon slices. According to Figure 5B,C, in various treatments of sturgeon slices, the groups significantly enriched (LDA > 5) at R0 included *Moraxellaceae* and *Acinetobacter*. The group significantly enriched at F0 included *Pseudomonas*. The significantly enriched groups in R12 included *Pseudomonadaceae*, *Pseudomonas*, *Gammaproteobacteria*, and *Proteobacteria*. Among them, the *Proteobacteria* had the highest LDA score at the phylum level, with a value of 5.27, while the genus *Pseudomonas* had the highest LDA score at the genus level, with a value of 5.6.

**FIGURE 6:**
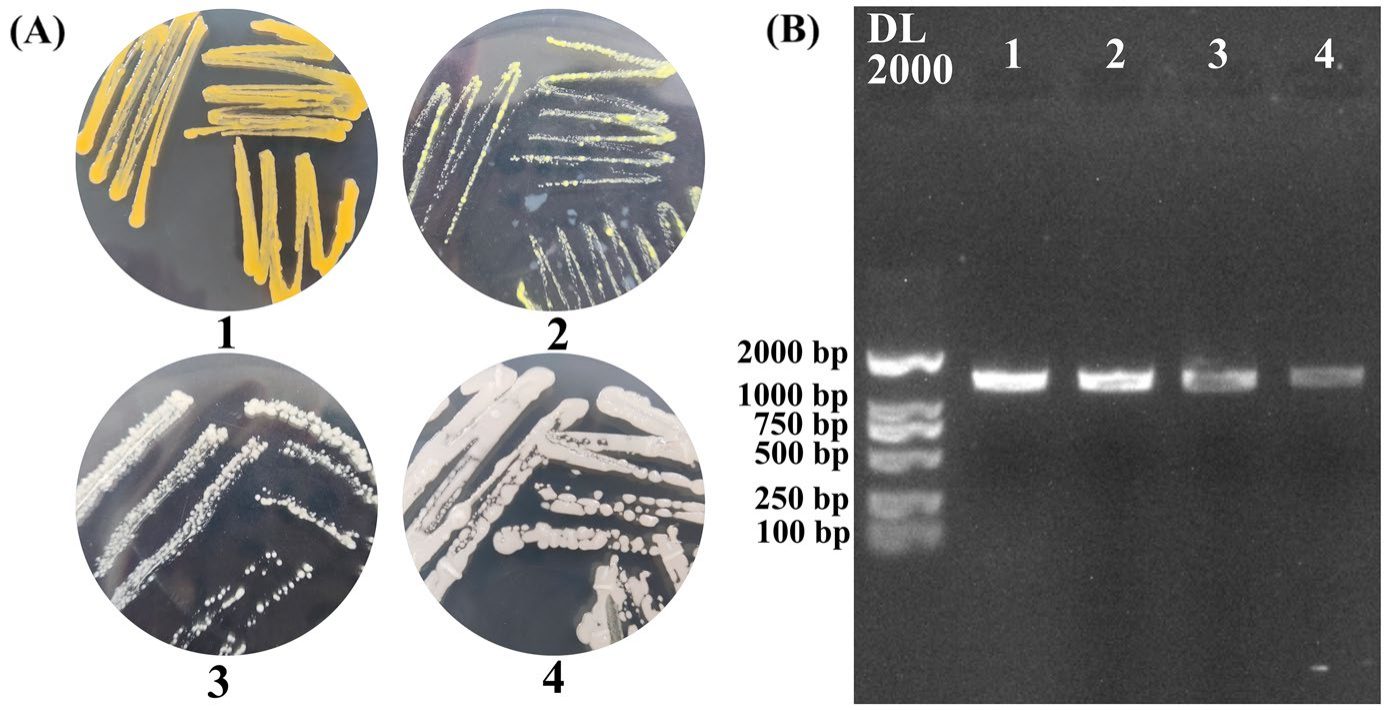
(A) Colony morphology of spoilage bacteria. (B) Electrophoretic map of PCR products of spoilage bacteria.

Samples were taken from sturgeon slices stored at ice temperature for 9–15 days and then cultured, isolated, and identified using different media. Four strains of spoilage bacteria with distinct differences were ultimately screened, and their colony morphology characteristics are shown in Figure 6A and Table S9. Subsequently, 16S rRNA PCR amplification and homology analysis were performed on the spoilage bacteria. From the electrophoresis image of the PCR amplification product, the band of spoilage bacteria was around 1500 bp in length (Figure 6B). Through comparison of homologous sequences in the NCBI database, the obtained homology was greater than 99.00%. Strain 1 had the highest sequence similarity with *Chryseobacterium* sp., reaching 99.93% (Table S10). The sequence similarity between strain 2 and *Microbacterium* sp. reached 100%; Strain 3 had the highest homology with *Empedobacter falsenii*, with a similarity of 99.78%; Strain 4 had the highest similarity of 100.00% with *Bacillus cereus* . It can be confirmed that strain 1 belongs to the *Bactericide*, strains 2 and 3 belong to the *Proteobacteria*, and strain 4 belongs to the *Firmicutes*. A total of four dominant spoilage bacteria were isolated from ice‐stored sturgeon fillets and identified as *Chryseobacterium* sp., *Microbacterium* sp., *Empedobacter falsenii*, and *Bacillus cereus* . Among these four bacteria, *Microbacterium* sp. and *Bacillus cereus* were more resistant to low temperatures. They had unique metabolic adaptations to break down nutrients in fish by secreting specific extracellular enzymes in the low‐temperature environment. The identification of these dominant spoilage bacteria provides a research basis for the in‐depth understanding of the spoilage mechanism of sturgeon fillets stored at ice temperature and the subsequent targeted development of efficient preservation strategies.

### 3.4 Analysis of Freshness Preservation Ability

**FIGURE 7:**
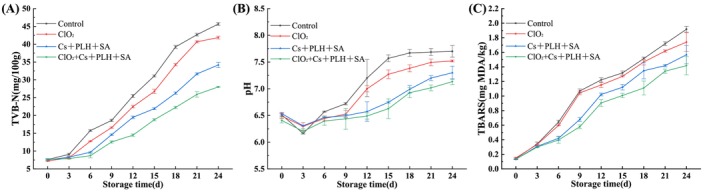
TVB‐N content (A), pH changes (B), and TBARS content (C) of sturgeon slices during ice temperature storage assisted by chlorine dioxide and coating.

In most cases, the TVB‐N value is an important indicator for evaluating the freshness of aquatic products (Chang et al. 2021). TVB‐N is closely related to the life activities of microorganisms and mainly produced through the degradation of proteins in fish meat by microorganisms. According to relevant regulations (GB 2733–2015 2015), when the TVB‐N in freshwater fish exceeded 20 mg/100 g, it reached the end point of spoilage. Therefore, we determined the freshness of fish slices by testing their TVB‐N values during storage. As shown in Figure 7A, the TVB‐N values of different treatment groups would increase with time. Compared to the control group, the ClO_2_ and Cs + PLH + SA treatment groups under ice temperature conditions were able to slightly and to some extent reduce the TVB‐N values in fish slices, respectively. Obviously, the ClO_2_ + Cs + PLH + SA treatment group had the lowest values. The time for the TVB‐N values of the control group, ClO_2_, Cs + PLH + SA, and ClO_2_ + Cs + PLH + SA treatment groups to reach 20 mg/100 g were 9–12 d, 9–12 d, 12–15 d, and 15–18 d, respectively. The method proposed in this article could extend the preservation time by at least 5 days compared to ice temperature storage alone. The results are in consistence with a previous study of Shahbazi et al. (2021). The change of pH is closely related to the content of TVB‐N in the growth process of microorganisms (Dubey et al. 2024), so the degree of fish fillet spoilage can be indirectly evaluated by the change in pH value. For this purpose, we tested the pH values of different treatment groups, and the results showed that the pH values of all groups did not differ among all treatments after processing and showed a trend of first decreasing and then increasing (Figure 7B). Similar results were seen in Chung et al. (2024) treatment of Atlantic Salmon fillets with ClO_2_. The decrease in pH value in the early stage of storage is due to the consumption of glycogen and the breakdown of adenosine triphosphate in sturgeon slices by the life activity of microorganism, resulting in the production of lactate and phosphate, and then leading to a decrease in pH value. As these components are completely consumed and microorganisms degrade the protein, the alkaline substances produced will cause an increase in the pH value of sturgeon slices. From the graph, the pH value of the fish fillet began to increase until the protein is completely decomposed after 3 days of storage. For different treatment methods, the increased rate of pH value was control group > ClO_2_ > Cs + PLH + SA > ClO_2_ + Cs + PLH + SA, indirectly proving that the last group had the strongest inhibitory effect on microorganisms. The thiobarbituric acid reactant value (TBARS) is related to the internal lipid auto oxidation process of fish meat. During the storage of aquatic products, the unsaturated fatty acids inside will undergo oxidation, producing substances such as malondialdehyde. The malondialdehyde produced can react with TBA to form a red complex, which can be used to determine the degree of rancidity of fish meat. From Figure 7C, the TBARS value continuously increased with storage time during the storage period of sturgeon. However, relatively speaking, the TBARS value of this group treated with ClO_2_ + Cs + PLH + SA increased the slowest, indicating that it had the best preservation effect on sturgeon slices and had a certain inhibitory effect on the oxidation of unsaturated fatty acids. Ali and Nassim (2023), who found that the GE‐XG + CN 1%, GE‐XG + BBE 7%, and GEXG + BBE 7% + CN 1% nanofibers delayed the lipid oxidation in raw peeled shrimps and extended their shelf life under cooled storage conditions for 6, 8, and 10 days, respectively. It can be seen from this that delaying lipid oxidation can extend the shelf life of fresh meat. The above study monitored the effect of different treatment groups on the ice temperature preservation of sturgeon slices from three aspects: TVB‐N, TBARS, and pH value. Finally, it was proved that the ice temperature preservation technology assisted by ClO_2_ + Cs + PLH + SA proposed in this paper exhibited the best preservation effect. It can extend the shelf life of fish fillets up to 15 days. From the above results, it can be seen that the change in TVB‐N value is a direct reflection of the degradation of fish protein, and its increase indicates that the microbial protease activity is enhanced, which leads to a decrease in the nutritional value of fish, while the increase of TBARS value is closely related to the oxidation of unsaturated fatty acids in fish, and the oxidized product not only affects the flavor of fish but also may produce harmful substances. Future research could focus on further optimizing the formulation of preservatives to reduce their potential impact on fish quality, as well as exploring the addition of natural antioxidants to synergistically inhibit fat oxidation and improve preservation.

The preservation work in this study was carried out in a laboratory simulation environment, which has certain differences from the actual storage environment. In the laboratory, we can control factors such as temperature, humidity, and packaging methods relatively precisely. However, in actual industrial preservation, the storage environment conditions are complex and changeable and cannot be precisely controlled. Meanwhile, Guizhou, where the experiment was conducted, is characterized by a high‐humidity environment and has significant environmental differences from regions such as the northwest of China. All of the above factors may have an impact on the actual industrial preservation applications. This study is only a preliminary exploration of ice temperature preservation of sturgeon fillets, and a detailed study on the interference of these actual environmental factors and the impact caused by regional differences has not yet been carried out. Therefore, in the promotion and application of industrial preservation, it is necessary to further evaluate the operability and effectiveness of laboratory results in industrial applications to improve the reliability and stability of preservation technology in industrial applications.

## 4 Conclusion

This article proposes an ice temperature preservation technology based on ClO_2_ and CS assistance, which could be applied to the storage of sturgeon slices. The optimal ratio obtained through response surface optimization was 3.07 g/100 mL NaCl, 3.25 g/100 mL glucose, and 4.05 g/100 mL sucrose, which could lower the freezing point of fish fillet to −2.50°C. It could not only lower the freezing point from −1.2°C to −2.57°C, but also inhibited the dominant spoilage bacteria in sturgeon slices. Through high‐throughput sequencing and traditional culture‐isolation techniques, it had been demonstrated that this preservation technology could significantly inhibit the growth and reproduction of microorganisms such as *Chryseobacterium* sp., *Microbacterium* sp., *Empedobacter falsenii*, and *Bacillus cereus* . The optimal preservation effect could be achieved when the concentrations of CS, sodium alginate, and poly‐L‐lysine HCl are 2.12, 0.72, and 1.02 g/100 mL, respectively. They can significantly reduce the content of TVB‐N and TBARS in fish meat and controlling the pH value. The ice temperature preservation technology assisted by chlorine dioxide and coating provides key reference factors for the storage and preservation of fish fillets and related aquatic products.

## Author Contributions

**Wenping Yang:** conceptualization (equal), formal analysis (equal), visualization (equal), writing – original draft (lead), writing – review and editing (lead). **Kunyu Sui:** data curation (equal), formal analysis (equal), investigation (equal), methodology (equal), visualization (equal), writing – review and editing (supporting). **Fawei Qiu:** investigation (supporting), methodology (supporting), visualization (supporting), writing – review and editing (supporting). **Qinhuizi Zhu:** investigation (supporting), methodology (supporting), visualization (supporting), writing – review and editing (supporting). **Jianlin Luo:** investigation (supporting), methodology (supporting), project administration (supporting), visualization (supporting), writing – review and editing (supporting). **Shirui Yu:** conceptualization (equal), data curation (equal), formal analysis (equal), funding acquisition (lead), investigation (equal), methodology (equal), project administration (lead), writing – review and editing (supporting).

## Conflicts of Interest

The authors declare no conflicts of interest.

## Supporting information


Data S1.

## Data Availability

The data that support the findings of this study are available on request from the corresponding author.
