# Supplementary material for: Application of Ice Temperature Storage Technology Assisted by Chlorine Dioxide and Chitosan for the Preservation of Fresh Fish Slices
Source: Food Sci Nutr. 2025 Apr 21;13(4):e70127. doi: 10.1002/fsn3.70127 (PMC12012004; doi:10.1002/fsn3.70127)
Supplement: Supplementary file 1 — Data S1. [file FSN3-13-e70127-s001.docx]

**Application of ice temperature storage technology assisted by chlorine dioxide and chitosan coating for the preservation of fresh fish slices**

Wenping Yang ^a^, Kunyu Sui ^b^, Fawei Qiu ^a^, Qinhuizi Zhu ^a^, Jianlin Luo ^b*^, Shirui Yu ^a,c*^

^a^ *Department of Food Science and Engineering, Moutai Institute, Renhuai 564507, China.*

^b^ *College of Biological and Environmental Engineering, Guiyang University, Guiyang 550025, China.*

^c^ *Guizhou Health Wine Brewing Technology Engineering Research Center, Moutai Institute, Renhuai 564507, China.*

^*^ *Corresponding author. E-mail addresses: ysr312004@126.com (S. Yu), bjfuljl@gmail.com (J. Luo)*


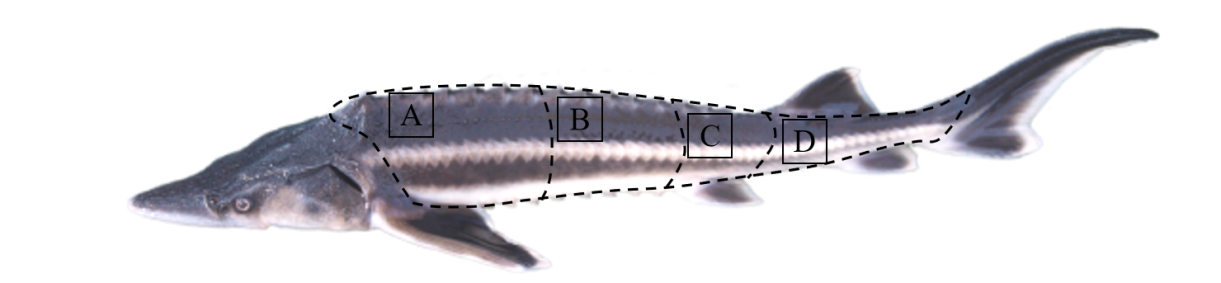


**Figure S1.** Schematic of sturgeon muscle segmentation.


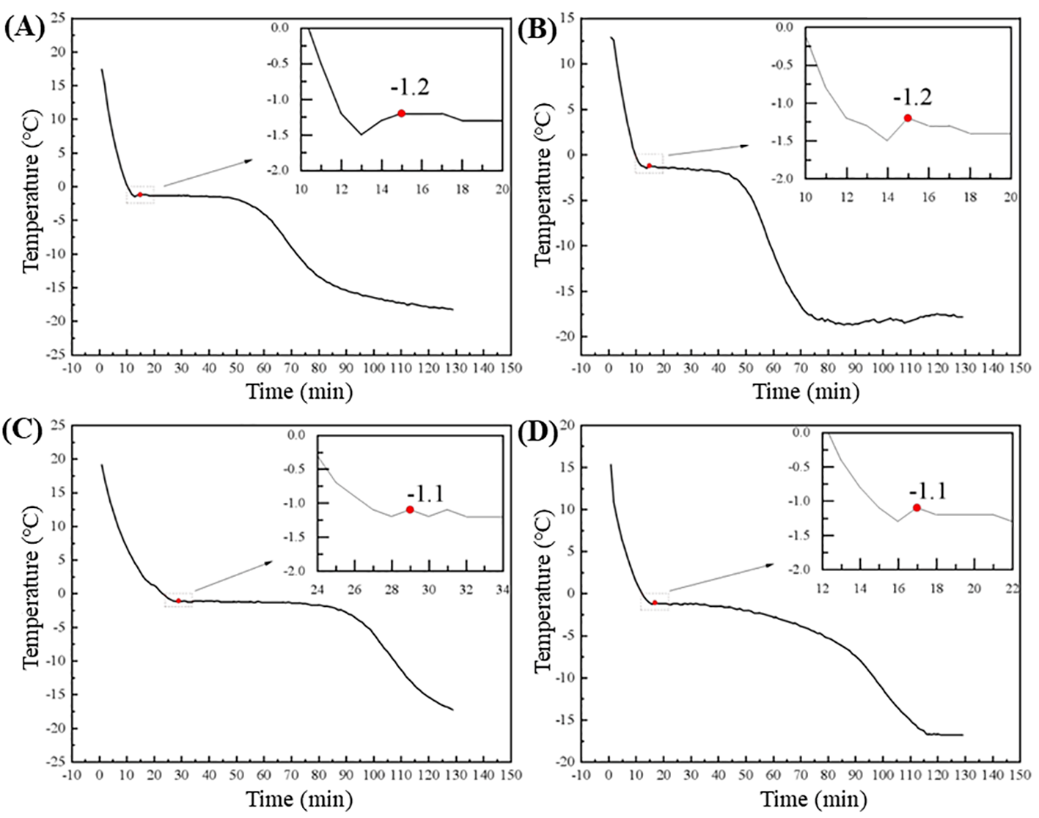


**Figure S2.** Freezing temperature curves of different body parts of sturgeon. (A) Front body, (B) middle body, (C) rear body, and (D) tail.


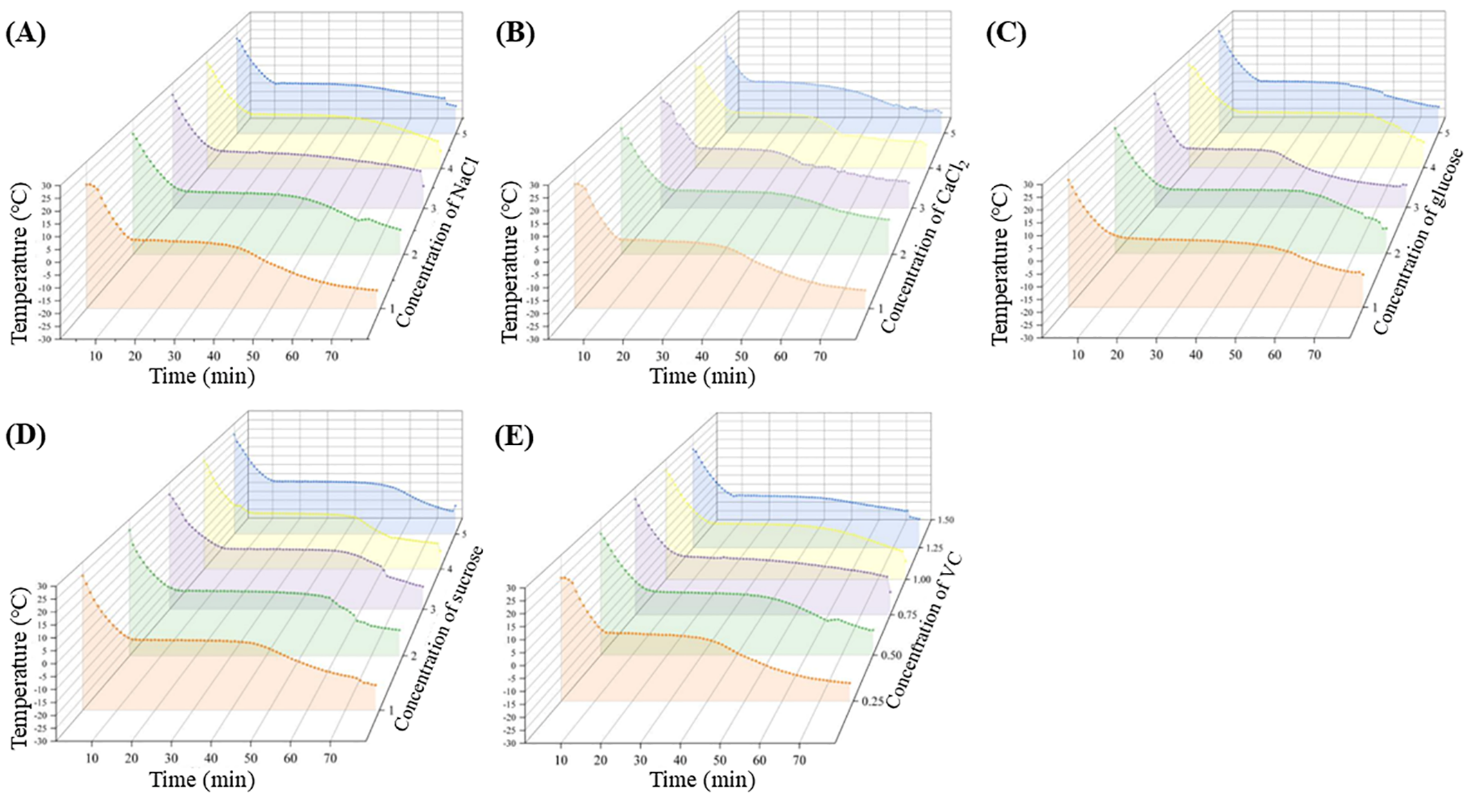


**Figure S3.** Freezing curves of sturgeon treated with different freezing point regulators. (A) NaCl, (B) CaCl_2_, (C) glucose, (D) sucrose, and (E) VC. The unit of concentration is g/ 100 mL.


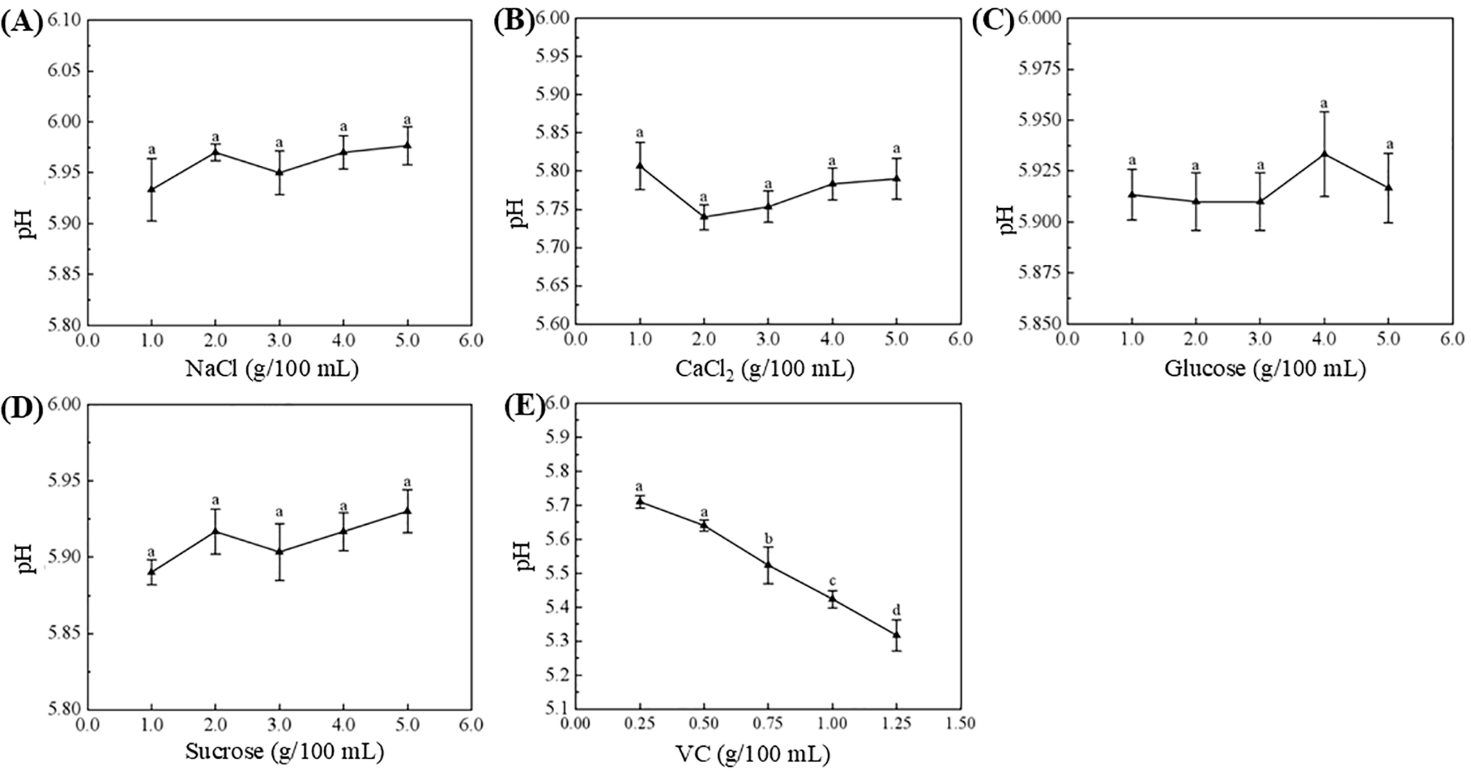


**Figure S4.** The effect of different freezing point regulators on the pH value of sturgeon. (A) NaCl, (B) CaCl_2_, (C) glucose, (D) sucrose, and (E) VC.


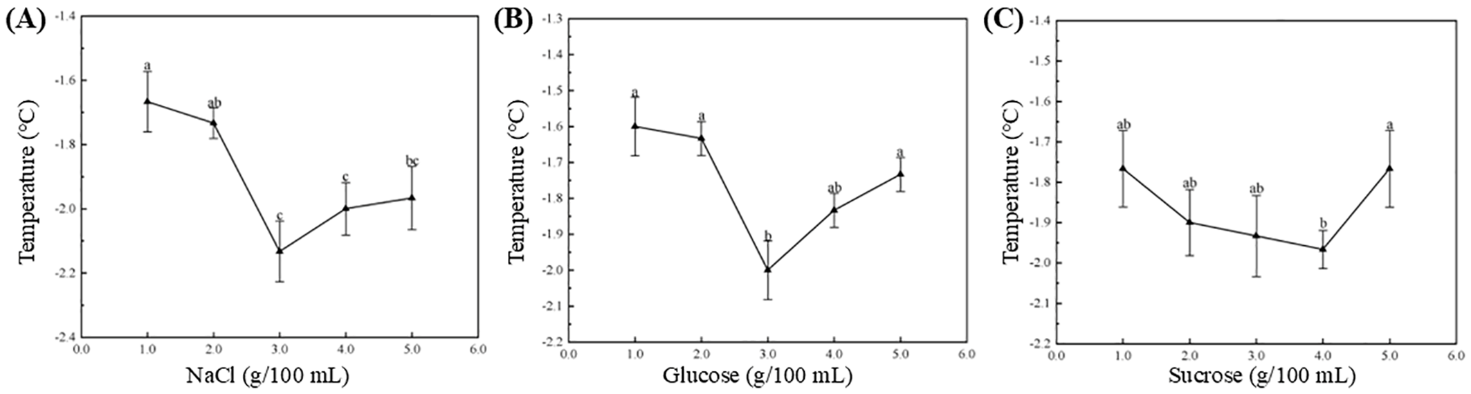


**Figure S5.** The effect of different concentrations of freezing point regulators on the freezing point of sturgeon. (A) NaCl, (B) glucose, and (C) sucrose.


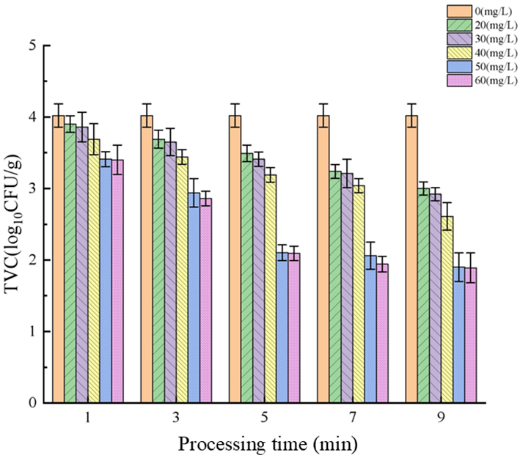


**Figure S6.** Antibacterial ability of chlorine dioxide with different concentrations.


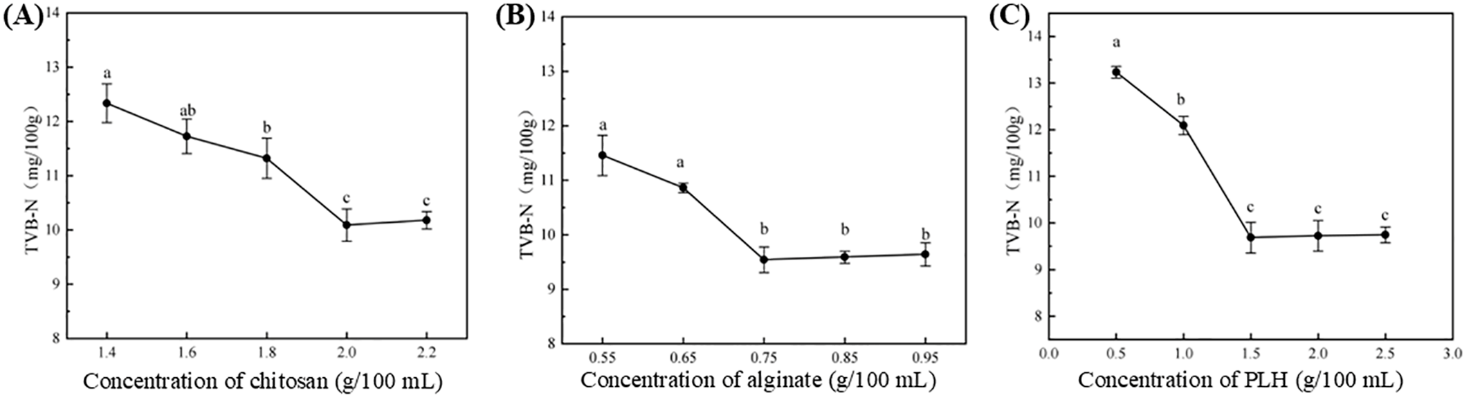


**Figure S7.** The TVB-N content of sturgeon slices treated with different concentrations of chitosan (A), alginate (B), PLH (C) after storage for 7 days.


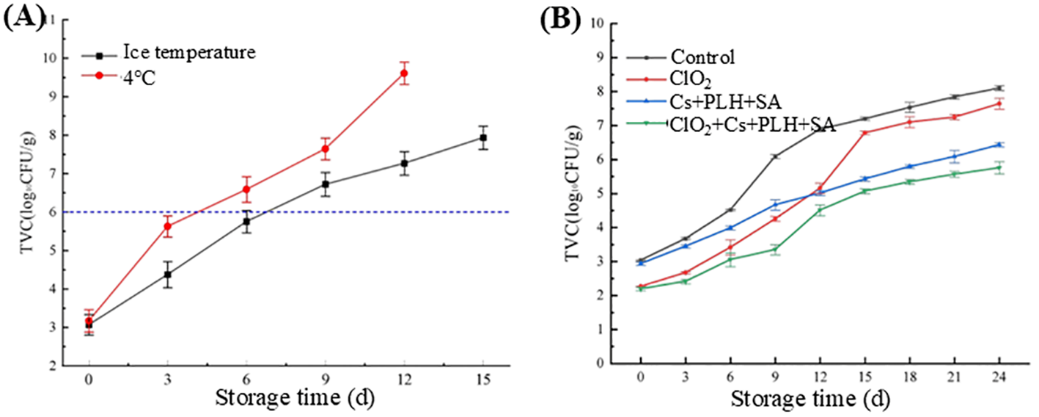


**Figure S8.** The impact of different treatment methods on the total number of bacterial colonies. (A) Ice temperature or 4℃. (B) Control, ClO_2_, Cs+PLH+SA, or ClO_2_+Cs+PLH+SA under ice temperature conditions.


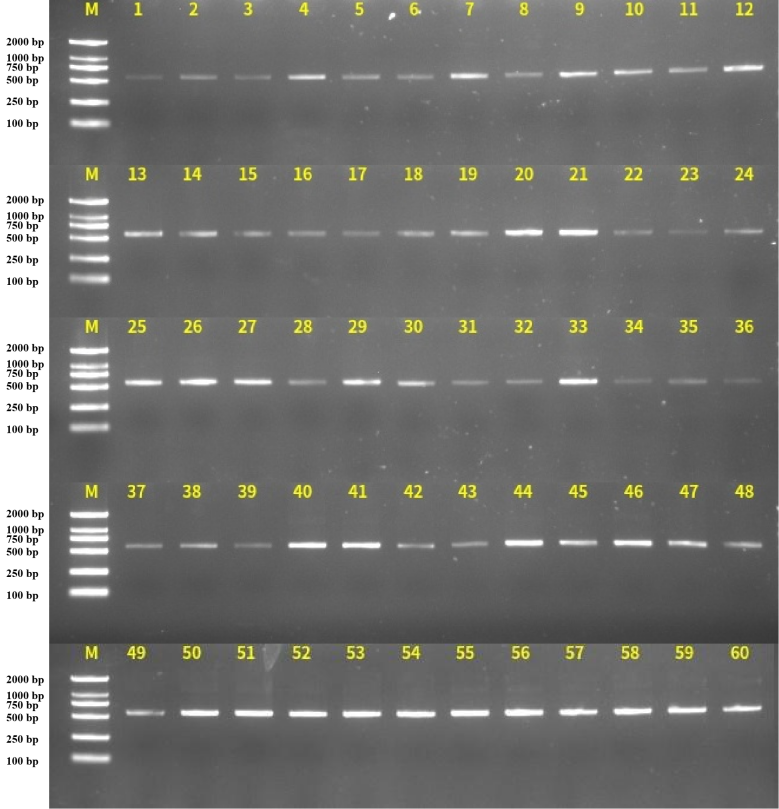


**Figure S9.** Electrophoretic images of PCR amplification products from different treatment groups. Ice temperature: 1-30, 4℃ refrigeration: 31-60.


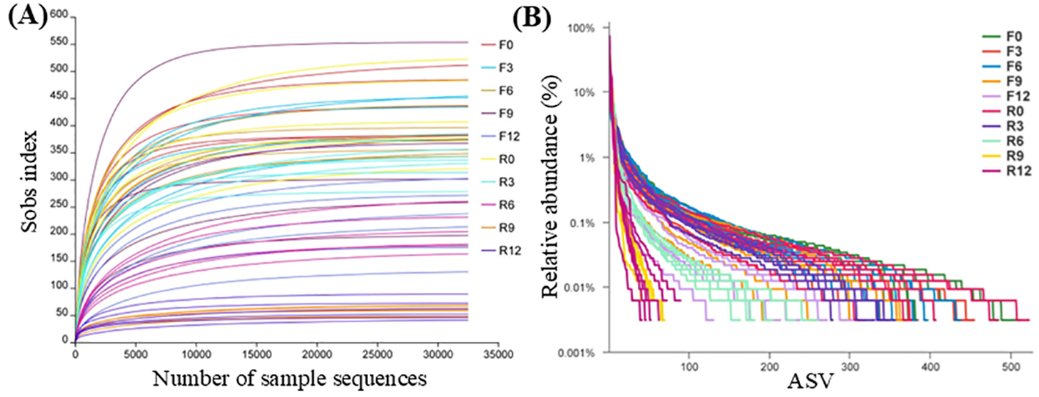


**Figure S10.** Dilution curve (A) and abundance (B) of microbial species.

**Table S1.** Experimental results of response surface optimization of freezing point regulator.

| Group | NaCl  (g/100 mL) | Glucose  (g/100 mL) | Sucrose  (g/100 mL) | Freezing point (℃) |
| --- | --- | --- | --- | --- |
| 1 | 3.0 | 2.0 | 3.0 | -1.8 |
| 2 | 3.0 | 3.0 | 4.0 | -2.6 |
| 3 | 3.0 | 4.0 | 5.0 | -2.1 |
| 4 | 4.0 | 3.0 | 3.0 | -1.9 |
| 5 | 3.0 | 2.0 | 5.0 | -2.0 |
| 6 | 2.0 | 4.0 | 4.0 | -2.0 |
| 7 | 3.0 | 3.0 | 4.0 | -2.6 |
| 8 | 4.0 | 3.0 | 5.0 | -2.1 |
| 9 | 2.0 | 2.0 | 4.0 | -2.2 |
| 10 | 3.0 | 3.0 | 4.0 | -2.6 |
| 11 | 4.0 | 2.0 | 4.0 | -1.7 |
| 12 | 3.0 | 4.0 | 3.0 | -2.1 |
| 13 | 4.0 | 4.0 | 4.0 | -2.4 |
| 14 | 2.0 | 3.0 | 5.0 | -2.0 |
| 15 | 3.0 | 3.0 | 4.0 | -2.5 |
| 16 | 2.0 | 3.0 | 3.0 | -2.1 |
| 17 | 3.0 | 3.0 | 4.0 | -2.5 |

**Table S2.** Results of the corresponding surface variance analysis.

|  | Sum of squares | Freedom | Sum of mean square | *F* value | *P* value | Significance |
| --- | --- | --- | --- | --- | --- | --- |
| Model | 1.34 | 9 | 0.1492 | 72.05 | < 0.0001 | *** |
| A-NaCl | 0.005 | 1 | 0.005 | 2.41 | 0.1642 |  |
| B-Glucose | 0.1012 | 1 | 0.1012 | 48.88 | 0.0002 | ** |
| C-Sucrose | 0.0112 | 1 | 0.0112 | 5.43 | 0.0526 |  |
| AB | 0.2025 | 1 | 0.2025 | 97.76 | < 0.0001 | *** |
| AC | 0.0225 | 1 | 0.0225 | 97.76 | 0.0132 | ** |
| BC | 0.01 | 1 | 0.01 | 4.83 | 0.064 |  |
| A^2^ | 0.2227 | 1 | 0.2227 | 107.53 | < 0.0001 | *** |
| B^2^ | 0.2738 | 1 | 0.2738 | 132.17 | < 0.0001 | *** |
| C^2^ | 0.3917 | 1 | 0.3917 | 189.09 | < 0.0001 | *** |
| Residual error | 0.0145 | 7 | 0.0021 |  |  |  |
| Omission item | 0.0025 | 3 | 0.0008 | 0.2778 | 0.8395 | not  significant |
| Pure error | 0.012 | 4 | 0.003 |  |  |  |
| Total error | 1.36 | 16 |  |  |  |  |

Note: “**” is *P*<0.01, “***” is *P*<0.001.

**Table S3.** Experimental results of response surface optimization of coating solution components.

| Group | Chitosan  (g/100 mL) | Alginate  (g/100 mL) | PLH  (g/100 mL) | TVB-N  (g/100 mL) |
| --- | --- | --- | --- | --- |
| 1 | 2.2 | 0.85 | 1.5 | 10.75 |
| 2 | 2.0 | 0.85 | 2.0 | 10.43 |
| 3 | 1.8 | 0.75 | 2.0 | 9.93 |
| 4 | 1.8 | 0.65 | 1.5 | 10.62 |
| 5 | 1.8 | 0.85 | 1.5 | 10.54 |
| 6 | 2.0 | 0.75 | 1.5 | 10.02 |
| 7 | 2.2 | 0.75 | 1.0 | 9.51 |
| 8 | 2.0 | 0.65 | 1.0 | 9.7 |
| 9 | 2.0 | 0.75 | 1.5 | 9.86 |
| 10 | 2.2 | 0.7 | 2.0 | 12.3 |
| 11 | 2.0 | 0.65 | 2.0 | 12.0 |
| 12 | 1.8 | 0.75 | 1.0 | 10.87 |
| 13 | 2.0 | 0.75 | 1.5 | 9.87 |
| 14 | 2.0 | 0.85 | 1.0 | 10.76 |
| 15 | 2.0 | 0.75 | 1.5 | 9.81 |
| 16 | 2.0 | 0.75 | 1.5 | 9.86 |
| 17 | 2.2 | 0.65 | 1.5 | 11.3 |

**Table S4.** Results of the corresponding surface variance analysis.

|  | Sum of squares | Freedom | Sum of mean square | *F* value | *P* value | Significance |
| --- | --- | --- | --- | --- | --- | --- |
| Model | 10.25 | 9 | 1.14 | 251.19 | < 0.0001 | *** |
| A- Chitosan | 0.4488 | 1 | 0.4488 | 99.03 | < 0.0001 | *** |
| B-Alginate | 0.1588 | 1 | 0.1588 | 35.04 | 0.0006 | ** |
| C-PLH | 1.82 | 1 | 1.82 | 402.17 | < 0.0001 | *** |
| AB | 0.0546 | 1 | 0.0546 | 12.04 | 0.0104 | * |
| AC | 3.48 | 1 | 3.48 | 767.69 | < 0.0001 | ** |
| BC | 1.73 | 1 | 1.73 | 382.75 | < 0.0001 | *** |
| A^2^ | 0.7532 | 1 | 0.7532 | 166.20 | < 0.0001 | *** |
| B^2^ | 1.03 | 1 | 1.03 | 227.57 | < 0.0001 | *** |
| C^2^ | 0.5012 | 1 | 0.5012 | 110.6 | < 0.0001 | *** |
| Residual error | 0.0317 | 7 | 0.0045 |  |  |  |
| Omission item | 0.0057 | 3 | 0.0019 | 0.29 | 0.8315 | not  significant |
| Pure error | 0.0261 | 4 | 0.0065 |  |  |  |
| Total error | 10.28 | 16 |  |  |  |  |

Note: “**” is *P*<0.01, “***” is *P*<0.001.

**Table S5.** High throughput sequencing data statistics of ice temperature group.

| Sample | Number of valid sequences | Number of bases | Average length |
| --- | --- | --- | --- |
| FA1 | 55599 | 23584693 | 424.19 |
| FA2 | 56753 | 24125771 | 425.10 |
| FA3 | 56634 | 24024821 | 424.21 |
| FA4 | 57871 | 24728216 | 427.30 |
| FA5 | 73850 | 31315857 | 424.05 |
| FA6 | 56693 | 24158473 | 426.13 |
| FB1 | 56771 | 24248323 | 427.13 |
| FB2 | 49008 | 20852304 | 425.49 |
| FB3 | 71408 | 30476757 | 426.80 |
| FB4 | 57259 | 24416982 | 426.43 |
| FB5 | 59851 | 25567113 | 427.18 |
| FB6 | 65068 | 27860172 | 428.17 |
| FC1 | 59458 | 25395177 | 427.11 |
| FC2 | 58301 | 24774267 | 424.94 |
| FC3 | 54587 | 23319052 | 427.19 |
| FC4 | 49699 | 21149661 | 425.56 |
| FC5 | 60138 | 25641605 | 426.38 |
| FC6 | 57996 | 24763368 | 426.98 |
| FD1 | 57556 | 24660709 | 428.46 |
| FD2 | 56994 | 24358493 | 427.39 |
| FD3 | 62384 | 26709818 | 428.15 |
| FD4 | 47245 | 19976053 | 422.82 |
| FD5 | 51547 | 21842638 | 423.74 |
| FD6 | 57411 | 24529834 | 427.27 |
| FE1 | 63777 | 27311647 | 428.24 |
| FE2 | 62089 | 26624431 | 428.81 |
| FE3 | 60703 | 26012672 | 428.52 |
| FE4 | 77091 | 32995641 | 428.01 |
| FE5 | 62500 | 26701825 | 427.23 |
| FE6 | 69233 | 29655587 | 428.34 |

Note: The second letters “A, B, C, D, E” represent the storage days of “0, 3, 6, 9, 12, and the numbers “1, 2, 3, 4, 5, 6” represent parallel experiments.

**Table S6.** High throughput sequencing data statistics of refrigerated group.

| Sample | Number of valid sequences | Number of bases | Average length |
| --- | --- | --- | --- |
| RA1 | 52369 | 22329337 | 426.38 |
| RA2 | 53350 | 22740317 | 426.25 |
| RA3 | 61479 | 26319912 | 428.11 |
| RA4 | 54580 | 23188653 | 424.86 |
| RA5 | 61552 | 25789077 | 418.98 |
| RA6 | 56355 | 23848693 | 423.19 |
| RB1 | 53496 | 22838468 | 426.92 |
| RB2 | 58537 | 25026443 | 427.53 |
| RB3 | 47908 | 20229575 | 422.26 |
| RB4 | 60980 | 26068587 | 427.49 |
| RB5 | 64308 | 27511799 | 427.81 |
| RB6 | 41777 | 17810910 | 426.33 |
| RC1 | 55390 | 23728734 | 428.39 |
| RC2 | 59796 | 25639508 | 428.78 |
| RC3 | 79851 | 34210082 | 428.42 |
| RC4 | 63397 | 27186637 | 428.83 |
| RC5 | 61587 | 26409857 | 428.82 |
| RC6 | 63521 | 27231030 | 428.69 |
| RD1 | 67435 | 28929284 | 429.00 |
| RD2 | 70026 | 30041389 | 429.00 |
| RD3 | 66714 | 28619191 | 428.98 |
| RD4 | 66137 | 28371864 | 428.99 |
| RD5 | 67938 | 29145487 | 429.00 |
| RD6 | 67211 | 28832923 | 428.99 |
| RE1 | 63106 | 27070884 | 428.97 |
| RE2 | 65607 | 28143877 | 428.98 |
| RE3 | 65471 | 28085419 | 428.97 |
| RE4 | 66219 | 28403112 | 428.93 |
| RE5 | 70291 | 30154068 | 428.99 |
| RE6 | 68630 | 29440292 | 428.97 |

Note: The second letters “A, B, C, D, E” represent the storage days of “0, 3, 6, 9, 12, and the numbers “1, 2, 3, 4, 5, 6” represent parallel experiments.

**Table S7.** Alpha diversity index of bacteria stored at 4℃.

| Sample | Richness index | | Diversity index | |
| --- | --- | --- | --- | --- |
|  | ACE | Chaol | Shannon | Simpson |
| RA | 415.88±28.99 ^a^ | 414.82±29.31 ^a^ | 3.99±0.33 ^a^ | 0.055±0.02 ^c^ |
| RB | 329.11±26.75 ^b^ | 328.1±25.97 ^b^ | 3.87±0.15 ^b^ | 0.055±0.01 ^c^ |
| RC | 207.27±34.89 ^c^ | 205.46±34.68 ^c^ | 2.42±0.1 ^c^ | 0.19667±0.03 ^b^ |
| RD | 58.37±8.61 ^d^ | 58.05±8.33 ^d^ | 1.86±0.21 ^d^ | 0.23833±0.05 ^b^ |
| RE | 60.91±15.89 ^d^ | 60.44±16.28 ^d^ | 1.7±0.32 ^d^ | 0.325±0.11 ^a^ |

Note: FA, FB, FC, FD, and FE represent storage at 4℃ for 0, 3, 6, 9, and 12 days, respectively.

**Table S8.** Alpha diversity index of bacteria stored at ice temperature.

| Sample | Richness index | | Diversity index | |
| --- | --- | --- | --- | --- |
|  | ACE | Chaol | Shannon | Simpson |
| FA | 431.24±54.27 ^a^ | 431.05±54.41 ^a^ | 4.46±0.19 ^a^ | 0.03167±0.01 ^c^ |
| FB | 410.33±41.65 ^a^ | 409.11±41.74 ^a^ | 4.23±0.23 ^a^ | 0.03667±0.01 ^c^ |
| FC | 378.77±32.35 ^a^ | 378.31±32.45 ^a^ | 4.28±0.29 ^a^ | 0.04333±0.02 ^bc^ |
| FD | 343.91±32.35 ^ab^ | 343.6±62.01 ^a^ | 3.69±0.65 ^b^ | 0.08667±0.05 ^b^ |
| FE | 226.262±58.3 ^b^ | 224.89±58.25 ^b^ | 2.525±0.48 ^c^ | 0.18333±0.07 ^a^ |

Note: FA, FB, FC, FD, and FE represent ice temperature storage for 0, 3, 6, 9, and 12 days, respectively.

**Table S9.** Morphological characteristics of bacterial colonies of spoilage bacteria.

| Strain | Colony color | Raised or flattened | Transparency | Surface | Texture |
| --- | --- | --- | --- | --- | --- |
| 1 | yellow | raised | non-transparent | glossy and smooth | viscous |
| 2 | light yellow | raised | semi-transparent | glossy and smooth | easily provoked |
| 3 | milky white | raised | non-transparent | glossy and smooth | easily provoked |
| 4 | white | flattened | non-transparent | matte and rough | waxy |

**Table S10.** Results of sequence homology alignment of spoilage bacteria in the ice temperature treatment group.

| Strain | Sequence length (bp) | Comparison results | Homology | Login Number |
| --- | --- | --- | --- | --- |
| 1 | 1392 | *Chryseobacterium sp.* | 99.93% | CP033918.1 |
| 2 | 1392 | *Microbacterium sp.* | 100.00% | OP314178.1 |
| 3 | 1386 | *Empedobacter falsenii* | 99.78% | AM238680.1 |
| 4 | 1425 | *Bacillus cereus* | 100.00% | MF179551.1 |
